# Accelerating the pace of ecotoxicological assessment using artificial intelligence

**DOI:** 10.1007/s13280-021-01598-8

**Published:** 2021-08-24

**Authors:** Runsheng Song, Dingsheng Li, Alexander Chang, Mengya Tao, Yuwei Qin, Arturo A. Keller, Sangwon Suh

**Affiliations:** 1 Bren School of Environmental Science and Management, University of California, Santa Barbara, Santa Barbara, CA 98121 USA; 2 University of Nevada, Reno, 1664 N Virginia St, Reno, NV 89557 USA; 3 Emory Rollins School of Public Health, 1518 Clifton Rd, Atlanta, GA 30322 USA

**Keywords:** Chemical toxicity, Environmental toxicity, Life cycle assessment, Machine learning, QSAR

## Abstract

Species Sensitivity Distribution (SSD) is a key metric for understanding the potential ecotoxicological impacts of chemicals. However, SSDs have been developed to estimate for only handful of chemicals due to the scarcity of experimental toxicity data. Here we present a novel approach to expand the chemical coverage of SSDs using Artificial Neural Network (ANN). We collected over 2000 experimental toxicity data in Lethal Concentration 50 (LC50) for 8 aquatic species and trained an ANN model for each of the 8 aquatic species based on molecular structure. The *R*^2^ values of resulting ANN models range from 0.54 to 0.75 (median *R*^2^ = 0.69). We applied the predicted LC50 values to fit SSD curves using bootstrapping method, generating SSDs for 8424 chemicals in the ToX21 database. The dataset is expected to serve as a screening-level reference SSD database for understanding potential ecotoxicological impacts of chemicals.

**Supplementary Information:**

The online version contains supplementary material available at 10.1007/s13280-021-01598-8.

## Introduction

Climate change, habitat losses and the exposure to various man-made chemicals are major threats to global biodiversity (Hartley 2002; Vörösmarty 2010; Malaj 2014). According to the Red List of Threatened Species by the International Union for Conservation of Nature (IUCN), 1256 out of the total 8455 threats are associated with pollution, of which 251 are due solely to the pesticide and herbicide (The IUCN Red List of Threatened Species).

Our understanding of chemical’s toxicity footprints on the ecosystem, however, is hampered by the sheer number and diversity of the chemicals used by the society, their wide variation in sensitivity across species, and the high costs—and therefore the scarcity—of experimental toxicity data (Bressler et al. 2006; Holmstrup 2010; Martin 2019). The number of unique chemicals have been produced or used in the European Union (EU) countries in excess of one tonne per year reached 15,000 in 2018 and is growing in the past years (ECHA Publishes Official Statistics for the Last REACH Registration Deadline). Different species may exhibit dramatically different sensitivity to the same chemical. Pyrethroid, for example, is extremely toxic to insects, but it is well tolerated by most mammals (Wolansky and Harrill 2008).

The Species Sensitivity Distribution (SSD) is an approach that allows estimating the potential ecosystem impacts of a chemical considering the variation in the sensitivity of species to toxicants. SSD uses the statistical distribution of toxicity data points (Lethal Concentration, or LC50, for example) across multiple species as a proxy measure for the ecotoxicological impact of single stressor to the entire community (forum, E.-U. E. 1998; Posthuma et al. 2001). SSDs, combined with an assessment factor, are often used in risk assessment to estimate the Predicted No Effect Concentration (PNEC) (Raimondo et al. 2008; Ping et al. 2011). In environmental risk assessment, PNEC is often regarded as the safe concentration for chemical under which the entire aquatic ecosystem is unlikely be adversely affected (Calow and Forbes 2003; Cunningham et al. 2009). Furthermore, SSD can be also used in Life Cycle Assessment (LCA) and Life Cycle Impact Assessment (LCIA), where the Hazard Concentration at which half of the species are adversely affected, or HC50 value, is often used to derive the ecotoxicity Characterization Factors (CFs) of chemicals (Rosenbaum 2008; Henderson 2011).

The challenge is that experimental toxicity data are scarce, and developing an SSD of a chemical requires multiple toxicity data points across multiple species (Garner et al. 2015). The recommended minimum sample size ranges from 8 to 15 (Newman et al. 2009; Lowry 2012). The ECOTOX database, one of largest databases for experimental toxicity values, contains about 500 organic chemicals with experimental toxicity data for aquatic species, and only about 80 aquatic species have been tested on more than 5 organic chemicals. In USETOX, which is one of the characterization models widely used in LCA, only about 2000 CFs, which can be calculated using SSD, were derived using experimental toxicity data that exist for organic chemicals (Rosenbaum 2008). The scarcity of experimental toxicity data is the primary barrier for developing SSDs and for understanding the ecotoxicological impact of chemicals (Andersen and Krewski 2009).

One of the approaches to overcome the scarcity of experimental data is the use of Quantitative Structure–Activity Relationship (QSAR) models. QSAR models estimate a chemical’s bioactivity or toxicity using the structure of the chemical in the absence of experimental data (Cherkasov 2014). QSARs often use linear regression or logistic regression models (Worth and Cronin 2003; Chen et al. 2012). Mayer and colleagues for example, predicted chronic toxicity of chemicals to multiple fish species using linear regression model and acute toxicity test data (Mayer et al. 1994). Raevsky and colleagues estimated the LC50 values of chemicals to Guppy, Fathead Minnow and Rainbow Trout using chemical similarity approach (Raevsky et al. 2008). These QSARs, however, are designed to be applied to targeted groups of chemicals such as those with nonpolar Mode of Action (MOA) (Raevsky et al. 2008), and, when applied to other groups of chemicals, fail to provide reliable predictions (Cherkasov 2014).

Recent progresses in machine learning techniques, however, opens an entirely new avenue of opportunities for developing predictive models (Haupt et al. 2008). Artificial Neural Network (ANN), for example, has been successfully applied to predict rate constants and reaction rates of chemicals in atmosphere (Allison 2016) and extreme weather (Liu, et al. 2016), and QSARs using simpler neural networks have also been used to estimate acute toxicity of chemicals to few aquatic species using inputs in varies formats. For example, Devillers developed QSAR model to estimate the acute toxicity of pesticide for *Lepomis macrochirus* (Devillers 2001). Martin and colleagues provided a new model in Neural Networks to estimate the LC50 (96 h) for *Fathead Minnow*, and achieved satisfying performance (Martin and Young 2001). However, because of the development of SSDs requires the ecotoxicity data in comparable experimental conditions applied across various taxa, therefore, the existing QSARs from different studies cannot be assembled together to generate SSDs, and currently there isn’t an established method to estimate SSDs with machine learning techniques. Researchers typically combine existing QSARs for multiple species, which may employ disparate machine learning models, limiting the interpretability of the results when used simultaneously to estimate SSDs. Furthermore, the existing QSARs were developed with various sizes of training dataset, which are often smaller than few hundreds of chemicals (Burden and Winkler 1999; Devillers 2001; Devillers 2001; Kaiser 2003).

In this study, we present a novel approach to develop SSDs for organic chemicals using assembled machine learning techniques, taking only molecular structure information as the input. We collected over 2000 experimental ecotoxicity data points in LC50, produced under comparable experimental conditions for 8 aquatic species. Using these data and molecular descriptors, we developed ANN models to estimate the ecotoxicity of chemicals in LC50. A total of 8 ANN models were trained on experimental toxicity data for each of 8 aquatic species: *Pimephales Promelas, Daphnia Magna, Oryzias Latipes, Oncorhynchus Mykiss, Lepomis Macrochirus, Cyprinodon Variegatus, Americamysis Bahia* and other water fleas. The performances of the predictive SSDs were evaluated on existing SSDs built by experimental data. The uncertainties of the ANN models as well as the predictive SSDs were analyzed. Finally, we applied our model and estimated the SSDs for over 8000 organic chemicals in the Toxicology Testing in the 21st Century (ToX21) database and characterized their SSDs as well as the HC5 values. The performances of log-normal, Gamma and Weibull distributions to fit SSD were also evaluated.

## Materials and Methods

### Ecotoxicity dataset collection

We collected 2521 experimental ecotoxicity data for non-ionizable organic chemicals on 8 aquatic species: *Pimephales Promelas, Daphnia Magna, Oryzias Latipes, Oncorhynchus Mykiss, Lepomis Macrochirus, Cyprinodon Variegatus, Americamysis Bahia* and other water fleas, from major public databases, including ECOTOX, eChem, EFSA and HSDB (Todeschini and Consonni 2008; eChemPortal-Home; ECOTOX|MED|US EPA; ESFA; Hazardous Substances Data Bank (HSDB)). Data from peer-reviewed literatures was also added as supplementary data source to develop the neural network models in this study (Russom et al. 1997; Devillers 2001; Martin and Young 2001; Raevsky et al. 2008; Results of ecotoxicity tests data conducted by Ministry of the Environment in 2014; Austin et al. 2015; Toropov 2017). The number of experimental data collected for each species can be found in Fig. S1 in supplementary information. To ensure data quality of the ecotoxicity dataset we collected from this study, the critical experimental conditions, such as the testing duration, chemical purity and *pH* values were strictly controlled. We filtered the datasets and used only the LC50 data with 96 h of duration for all species except for water fleas, for which 48 h’ data was used due to the concerns of dataset size. Chemical purity must be higher than 85%. And the *pH* value must be in the range of 5 to 9. Experimental data that not meet these requirements was discarded. For chemical with multiple experimental values, the geometric mean was used in the final dataset. To utilize some of the discarded data, and to increase the diversity of the species taxa, experimental values that met our data selection procedure for other water fleas in ECOTOX database was combined and treated as an individual species in this study. Within this category, there are 20 chemicals for *Ceriodaphnia Dubia,* 13 chemicals for *Daphnia Pulex and* 63 chemicals for Mix Water Flea (not specified). Additional information, such as the CAS number, SMILEs, molecular weight and the chemical names were also collected, for referencing purpose. The unit of the LC50 values were converted to log10(LC50) in μmol/L. The final dataset is available in the supplementary information. mol/L. The final dataset is available in the supplementary information.

### Two-step molecular descriptor selection

The original molecular structural descriptors were calculated using Python packages rdkit and Mordred (rdkit: The official sources for the RDKit library. 2017; Moriwaki et al. 2018). The descriptor calculators can produce over 2000 descriptors for a single chemical, including basic physicochemical properties and autocorrelation descriptors. Large amount of descriptors could lead to overfitting problem (Kaiser 2003; Cherkasov 2014). Two-step feature selection procedures: filter-based plus tree-based feature selection, were used in this study to extract more meaningful descriptors (Guyon and Elisseeff 2003; Saeys et al. 2007).

Filter-based feature selection removes descriptors that have low variance, as well as the descriptors have high mutual correlations with others (Stojić et al. 2010). Tree-based feature selection method ranks the importance of each descriptor by their contribution to the prediction results in a decision tree model (Sugumaran et al. 2007) (Broderius and Kahl 1985). In this study, during the filter-based feature selection, descriptors with variance lower than 10 were discarded. Then, the correlations between every leftover descriptor were calculated and the second descriptor was discarded if a descriptor pair has correlation higher than 0.6. A decision tree regressor in Python package Sklearn was used as the basis for the tree-based feature selection on the remaining descriptors (Pedregosa 2011). The descriptors that contribute to the toxicity endpoint three times higher than the mean contribution were selected as the final descriptors in this study. As a result, the final descriptors are same for every chemical for one species, but are different between species (different ANN models). In this study, we used 8 to 15 structural descriptors for developing our models. The most frequently utilized molecular descriptor was SLogP (Wildman–Crippen LogP), which appeared in all models. Xp-2dv (2-ordered Chi path weighted by valence electrons) and PEOE_VSA6 (MOE Charge VSA Descriptor 6) were used in more than 3 models. The full list of descriptors used to develop each model in can be found in Table S6 of the supplementary information.

### The development of neural networks models and their applicable domain

ANNs were used as the modeling basis of the QSARs in this study. The ANNs were developed using Tensorflow and Keras in Python 2.7 (Chollet 2015; Abadi et al. 2016). The hyper-parameters of ANNs that were optimized through fivefold cross-validation in this study, including the number of hidden layer(s), the number of hidden neuron(s) in each layer, the regularization factor and the type of activation function. These hyper-parameters were optimized by minimizing the mean square error (MSE) of the ANN models while holding others constant. The final models were built using the hyper-parameters that generated the lowest MSE during cross-validation. The final model performances were reported on 20 chemicals that were randomly selected and left out during model development for each species. The ANNs were built on the rest of data.

ANNs have better performance on inputs that are like the training data. We used Euclidean distance from the input descriptors to the centroid of our training data as the metric to evaluate the Applicable Domain (AD) in this study. The Euclidean distance is calculated as:

1 $$d_{n} = \sqrt {\sum \left( {X_{i} - C_{i} } \right)^{2} }$$

where $d_{n}$ is the distance of chemical *n* to the centroid of training data *C*; $X_{i}$ and $C_{i}$ are the *i*th molecular descriptors of the input chemical and the training data. The centroid of the training data was calculated as the mean value of the molecular descriptors of all chemicals in the training data.

Whether an input chemical falls inside the model AD was determined by comparing a threshold value *K* with the distance $d_{n}$. For each ANNs, we first selected an initial *K* and then grouped the chemicals in the validation dataset by their distance to the centroid of the training data comparing with the *K* value (smaller or larger). The differences of the MSEs between these two groups were calculated. We then gradually increased the *K* value. The MSE differences changed accordingly since the chemicals within each group are different. We selected the *K* value that has the largest MSE difference to be the final threshold for model AD. The performance of this AD estimation was reported on the chemicals in the testing dataset.

### The development of SSDs and their uncertainties

SSD is a statistical distribution that illustrate the variation in the response of species to the exposure of chemicals. The development of SSD begins with the generation of individual toxicity value of chemicals to species. In this study, we used LC50 values of chemicals to aquatic species. The LC50s are ranked from low to high, or the most sensitive to the least sensitive species. On the SSD graph, as shown on Fig. 2, the *x*-axis is the concentration of chemical, and the *y*-axis stands for the percentage of species affected. For each data point, the location on *y*-axis is the Median Rank position of it. Which is calculated using the ppoint function in R, and reproduced in Python (ppoints function|R Documentation).

Therefore, the LC50 values are used to estimate the Cumulative Distribution Function (CDF) of a selected distribution. Most of the SSDs were fitted using normal or log-normal distributions (Wheeler et al. 2002; Aldenberg and Rorije 2013). Other statistical method including log-logistic distribution and Burr Type III method are also exist but have not been widely used (Aldenberg and Slob 1993; Wheeler et al. 2002). In this study, we used log-normal distribution as the basic distribution to fit SSDs, which is justified by the OVL analysis. The CDF of log-normal distribution is presented in Eq. (2):

2 $$F_{x} \left( x \right) = ~\Phi \left( {\frac{{\left( {\ln x} \right) - ~\mu }}{\sigma }} \right)$$

where $\Phi$ is the CDF for a standard normal distribution *N*(0, 1), shown in Eq. (3), and $\mu$ and $\sigma$ are the mean and standard deviation.

3 $$\Phi \left( x \right) = ~\frac{1}{{\sqrt {2\pi } }}~~e^{{ - \frac{1}{2}x^{2} }}$$

In this study, the decision of using log-normal distribution to fit SSD was made through running Overlapping Coefficient Analysis (OVL) testing on the screening results of ToX21 database. OVL is a measurement for the similarity of distributions, which compare the percentage of overlapping of the Probability Density Function (PDF) (Qin and Suh 2017). Equation (4) shows the mathematic representation of OVL for distributions $f_{a} \left( x \right)~$ and $f_{b} \left( x \right)$:

4 $$\Delta \left( {f_{a} \left( x \right),~f_{b} \left( x \right)} \right) = ~\smallint {\text{min}}\{ f_{a} \left( x \right),~f_{b} \left( x \right)\} ~{\text{d}}x~$$

For each chemical in ToX21 library, the actual distribution of the LC50 values on 8 species were compared with the empirical distributions that are fitted using the mean and standard deviation values on log-normal, Weibull and Gamma distributions. The area of overlapping was calculated.

Bootstrapping approach was used to estimate the uncertainty of SSD due to the limited amount of data points (MacKinnon et al. 2004). During each iteration of bootstrapping, eight data points were resampled using the fitted distribution curve and the newly sampled data points were used to construct new distribution curve. This process was repeated for 1000 times, generating the upper and lower bounds of SSD for each chemical. The uncertainty of the QSAR predictions were also considered in the SSDs. Depending on whether the chemical fell inside or outside a model AD, different MSEs were attached to the QSAR predicted values. Therefore, the upper and lower bounds of SSDs can be reported.

### Database screening

The chemical list in the ToX21 project is used as the candidates to be screened against the models developed in this study (US EPA 2015a). ToX21project aims to develop better toxicity assessment techniques in high-throughput robotic screening system. To date, about 10 000 chemicals have been tested under the project, and the screening results help to identify chemicals for further investigation (US EPA 2015a). We removed inorganic chemicals, ionized chemicals and chemicals that can’t find SMILEs within this list. As a result, 8424 chemicals are left and developed predictive SSDs using the models in this study. Among these chemicals, 1239 chemicals fell into the ADs for more than 4 (out of 8) ANN models. We considered these predictive SSDs are trustful and discarded the rest of predictive SSDs.

HC5 values for these (1239) chemicals were derived from the predictive SSDs. Among them, 218 chemicals were registered in the ECHA database, therefore we were able to find the production bands for them (Registered substances-ECHA). To consider ecotoxicity and production volume at the same time when comparing chemicals, we considered them when evaluating the threatening of the candidate chemicals. The threatening is calculated as described in Eq. (5). The screening results for all chemicals, as well as their production band can be found in the supporting information.

5 $$T = ~\frac{P}{{HC5}}$$

where *T* stands for the threatening (tonne·L/year·umol), which is a comparative score; $P$ (tonne/year) is the annual production band reported in ECHA database; $HC5$ (umol/L) is the hazardous concentration read from the predictive SSD.

## Results

### ANN model performances and applicable domain

**Fig. 1:**
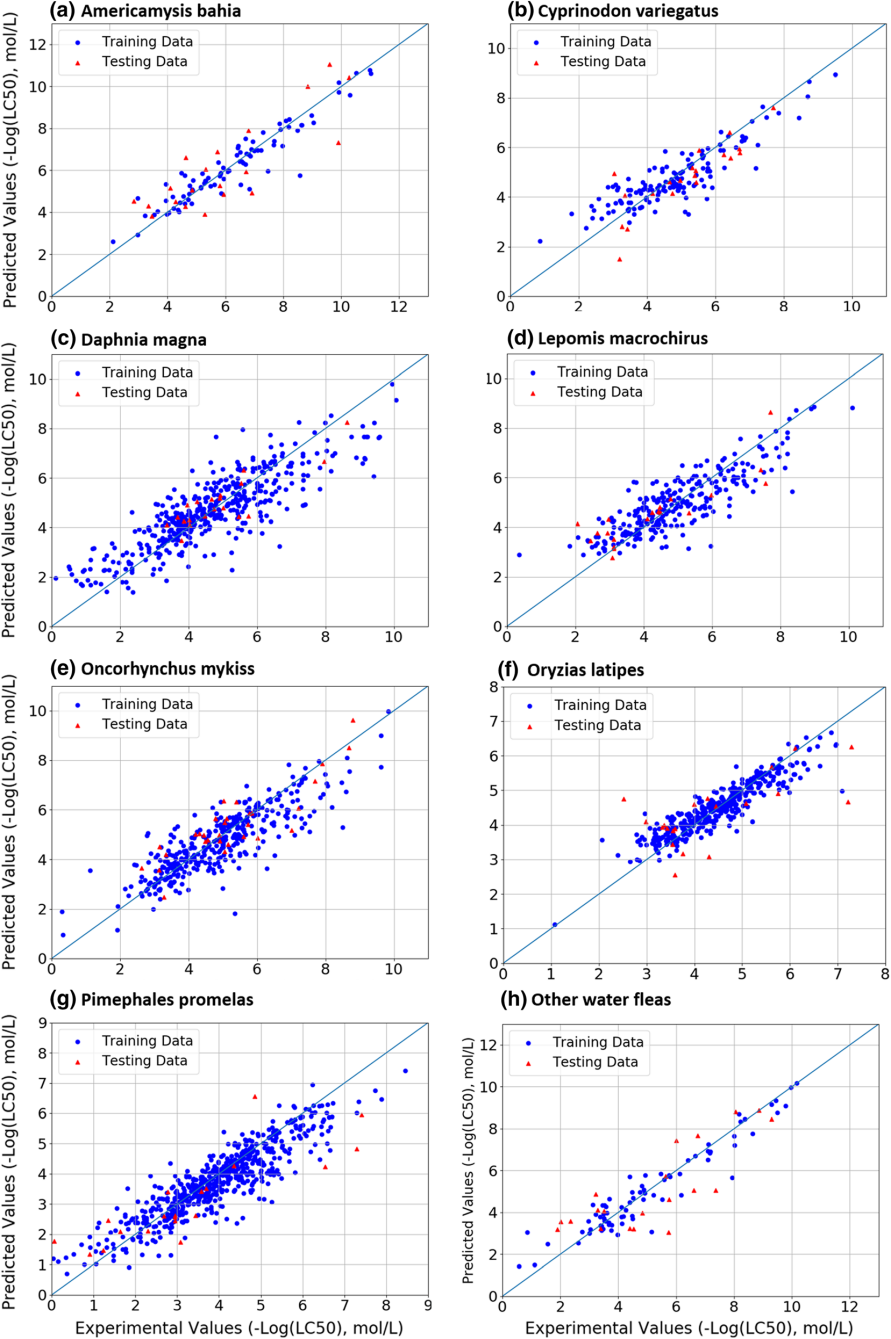
The performances of all models in this study on the training data (blue circles) and testing data (red triangles). The horizontal axis is the experimental values, and the vertical axis is the predicted values. The model structures were tuned using cross-validation technique

Figure 1 shows the performance of the ANN models. Circles in blue color are the training data points and triangles in red are randomly selected testing data. If predicted values match perfectly with experimental values, all the data points would be perfectly aligned on the line, *x* = *y*. The *R*^2^ of these ANN models ranged from 0.54 to 0.75 (mean 0.67, medium 0.69) on the testing data. More information about the hyper-parameters of these models, such as the number of hidden layers and neurons, is summarized in Table 1. Other details about the model structure, including the activation functions and regularization factors during training can be found in the supplementary information (Table S1). According to the results on the testing chemicals, the models for *Daphnia Magna* and *Oncorhynchus Mykiss* showed the highest *R*^2^ on testing data (0.75), followed by the *Lepomis Macrochirus* (0.72) and *Pimephales Promelas* (0.71) models, while the *Oryzias Latipes* model showed the lowest R^2^ on the testing data (0.54).

**Table 1:** The performance of the ANN models on the testing data for the 8 aquatic species in R^2^. The number of hidden layers and hidden neurons for each ANN model

	*PP	*DM	*OL	*OM	*LM	*CV	*AB	*OWF
Model performance (R^2) on testing data	0.71	0.75	0.54	0.75	0.72	0.66	0.67	0.63
Number of hidden layer	2	1	2	2	2	2	1	2
Number of hidden neuron in each layer	32 × 16	16	64 × 32	64 × 32	32 × 16	16 × 8	16	16 × 8

*Spcies acronyms: *Americamysis Bahia* (A.B.); *Daphnia Magna* (D.M.); *Lepomis Macrochirus* (L.M.); *Oncorhynchus Mykiss* (O.M.); *Cyprinodon Variegatus* (C.V.); *Oryzias Latipes* (O.L.); *Pimephales Promelas* (P.P.) and Other Water Fleas (O.W.F.)

To evaluate the model prediction confidence, we employed the concept of Applicable Domain (AD) to characterize the prediction accuracies of the ANN models and serves as a proxy to estimate whether a chemical is appropriate for the QSARs. The results of AD analysis are presented in Table S3 in the supplementary information. Among the ANN models that we developed, *Oncorhynchus Mykiss* and the *Lepomis Macrochirus* models have the narrowest ADs. For these two models, the mean square error (MSEs) of the testing data inside of the ADs was 6%, while those outside of AD were 15% and 22%, respectively. For the *Pimephales Promelas* model, however, the average MSEs inside and outside of AD were 8% to 220%, respectively, indicating limited utility of the model outside of AD.

### Predictive species sensitivity distributions and evaluations

Using our ANN models, we estimated the LC50 values for 8424 chemicals from the ToX21 database for each of the 8 aquatic species. We also estimated the prediction errors of the ANN models, as well as the inherent error of SSDs due to the limited number of data points. These SSDs can be found in the supporting information. Given the large number of chemicals in our results, we randomly selected a few chemicals to compare our predictive SSDs with the SSDs derived from experimental data. Elaborated here is one of them, DCMU (*3-(3,4-dichlorophenyl)-1,1-dimethylurea*), an algaecide.

**Fig. 2:**
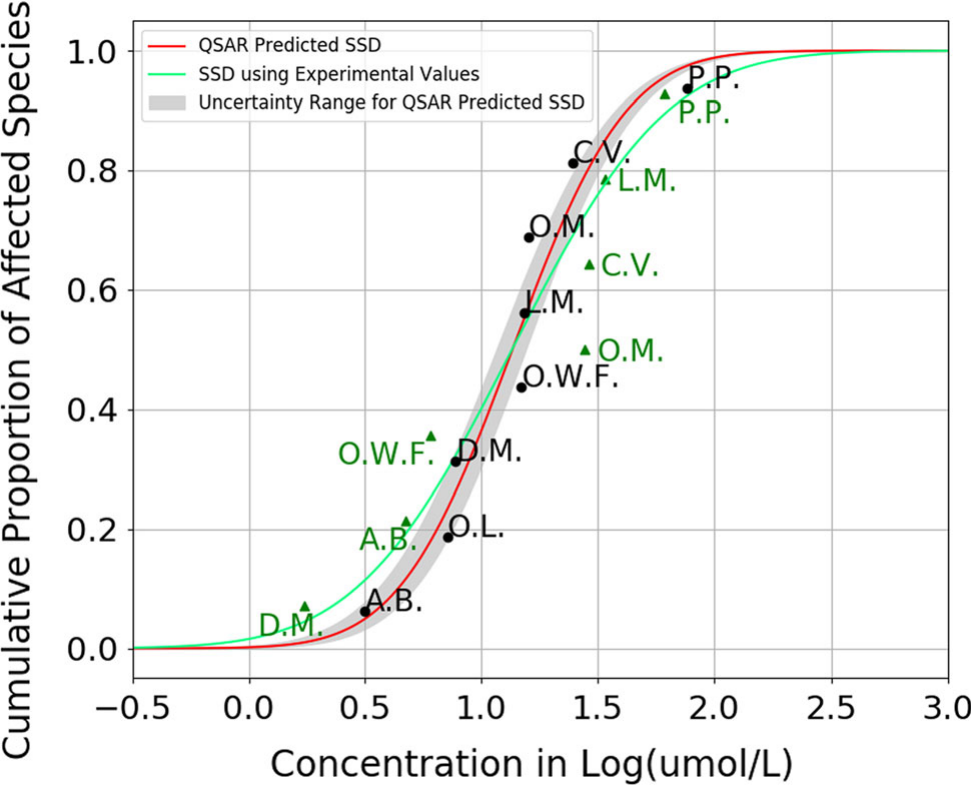
The SSD of DCMU (solid red line) constructed using the ANN-based LC50 values (black points), along with the uncertainty of ANN predictions (gray area), based on the model AD estimation for *Americamysis Bahia* (A.B.), *Daphnia Magna* (D.M.), *Lepomis Macrochirus* (L.M.), *Oncorhynchus Mykiss* (O.M.), *Cyprinodon Variegatus* (C.V.), *Oryzias Latipes* (O.L.), *Pimephales Promelas* (P.P.), and Other Water Fleas (O.W.F.). The SSD in green was constructed using experimental LC50 values found for 7 species)

The predictive SSD for DCMU is shown in red line in Fig. 2. The figure also shows the uncertainty range of the ANN-derived SSD in gray. This uncertainty range was calculated using the prediction error of each ANN model, which was determined by whether this chemical fell into the AD of each model or not. For a comparison, we collected experimental data for the same species, and we located experimental LC50 values for the same list of species other than *Oryzias Latipes*, which were unavailable in the literature and databases available to us. Using these experimental values, we constructed an SSD as shown by the green line in Fig. 2. According to the SSD derived from experimental values, the HC5 of DCMU is about 1.82 mmol/L, whereas the HC5 from the ANN-based SSD ranged from 2.51 to 3.24 mmol/L. Both experimental SSD and the predictive SSD show that *Pimephales Promelas* has the best tolerance to DCMU in water, with an experimental LC50 of 61.7 mmol/L and a predicted LC50 of 75.9 mmol/L. Figure 2 indicates that the predicted SSD tends to show lower toxicity for this chemical at lower concentration (i.e., < 0.5 Log μmol/L), and higher toxicity with higher concentration (i.e., > 1.5 Log μmol/L), which will be discussed in the next section.

Another 10 organic chemicals were randomly selected from the ECOTOX databases to evaluate the SSDs derived from our ANN models. We collected experimental LC50 data of these chemicals on other species than the aforementioned 8 species in order to avoid any overlap with the training data we used to develop our ANN models. Given the inherent uncertainty in SSDs due to the limited number of data points, we used the bootstrapping technique to visualize the potential range of SSDs. The mean, lower, and upper bounds of HC50 (hazardous concentration for 50% of the species) values on both predictive and experimental SSD curves are presented in Table 2. The Overlapping Coefficient (OVL) score in Table 2 shows the percentage of overlapping of the area of the predictive distribution and the experimental distribution. The detailed model prediction data for each of the chemicals, as well as the experimental LC50 values can be found in Table S4, and in the supplementary information. The predictive SSD, experimental SSD along with their overlapping area for chemical *chlorpyrifos* (*2921-88-2*) are presented in Fig. S2 as an example.

**Table 2:** The HC50 values of 10 chemicals in the ECOTOX database, along with the mean HC50 values for both ANN-based SSD and the experimental SSD, as well as the percentage of overlapping of the distributions based on the predictive and experimental SSDs

Chemical CAS	Chemical name	HC50 mean (lower, upper bounds) in log (μmol/L)		OVL score (%)
		Predicted	Experimental	
50-29-3	*Clofenotane*	− 0.45 (− 1.5, 0.62)	− 0.85 (− 1.43, − 0.26)	70.6
87-86-5	*Pentachlorophenol*	0.32 (0.04, 0.62)	0.23 (− 0.11, 0.54)	89.6
58-89-9	*Lindane*	1.29 (0.26, 2.22)	0.87 (0.36, 1.4)	65.8
60207-90-1	*Propiconazole*	0.64 (0.08, 1.25)	0.88 (0.5, 1.25)	75.9
138261-41-3	*Lmidacloprid*	2.1 (1.4, 2.8)	1.65 (0.66, 2.7)	77.6
115-29-7	*Endosulfan*	− 0.46 (− 1.09, 0.23)	− 0.99 (− 2.12, 0.1)	72.0
2921-88-2	*Chlorpyrifos*	− 0.03 (− 0.63, 0.66)	0.01 (− 0.76, 0.84)	92.0
206-44-0	*Fluoranthene*	0.9 (0.22, 1.58)	0.23 (− 0.04, 0.54)	50.3
62-53-3	*Aniline*	2.48 (2.21, 2.76)	2.71 (2.04, 3.42)	55.2
333-41-5	*Diazinon*	0.1 (− 0.72, 0.91)	0.04 (− 0.81, 0.87)	96.8

Table 2 shows that the predicted HC50 values generated by the ANN models are generally in line with the experimental SSDs. The OVL results show that 8 out 10 chemicals have OVL score higher than 70%, which means that 70% of the area in the predictive SSD overlap with the SSD generated by the experimental data. Among them, the predictive SSD for the chemical *diazinon* (333-41-5) shares the largest overlapping area with the experimental SSD (96.8%), followed by the chemical *chlorpyrifos* (2921-88-2) by 92.0% overlapping area. The predictive SSD shows the lowest OVL score is the one for chemical *fluoranthene* (206-44-0) with OVL score 50.3% and followed by the SSD for chemical *aniline* (62-53-3) with OVL score 55.2%.

**Fig. 3:**
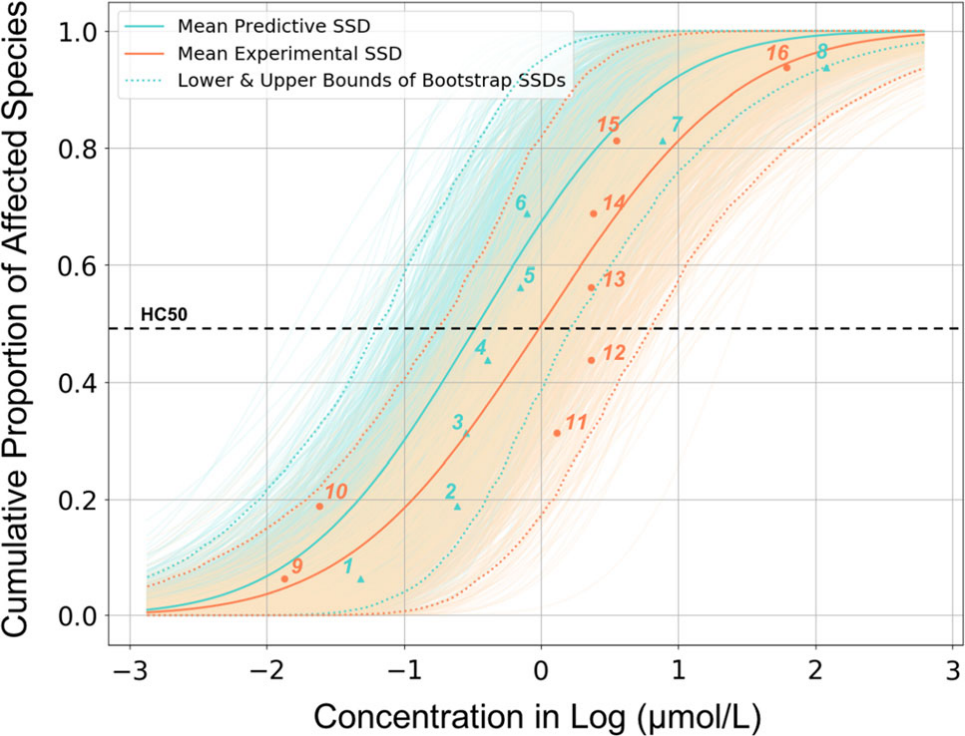
The mean (solid blue line), upper (97.5%), and lower (2.5%) bounds (dash blue lines) of the predictive SSD, and the mean (solid red line), upper (97.5%), and lower (2.5%) bounds (dash red lines) of the experimental SSD for *chlorpyrifos*. Each data point and numbers on the curves represent a species for corresponding data group (predictive, blue, or experimental, red). 1: *Americamysis Bahia* (Crustaceans, shrimp); 2: *Cyprinodon Cariegatus* (Fish); 3: *Daphnia Magna* (Crustaceans, water flea); 4: *Lepomis Macrochirus* (Fish); 5: *Pimephales Promelas* (Fish); 6: *Oncorhynchus Mykiss* (Fish); 7: *Oryzias Latipes* (Fish); 8: Other water fleas (Crustaceans, water flea); 9: *Pungitius Pungitius* (Fish); 10: *Gasterosteus Aculeatus* (Fish); 11: *Neocaridina Denticulate* (Crustaceans, shrimp); 12: *Lctalurus Punctatus* (Fish); 13: *Aplexa Hypnorum* (Molluscs); 14: *Carassius Auratus* (Fish); 15: *Zilchiopsis Collastinensis* (Crustaceans); 16: *Sialis Lutaria* (Insects/Spiders)

We used the 97.5% percentile and the 2.5% percentile as the upper and lower bounds, respectively, of the 1000 time bootstrapping when fitting LC50 values to SSDs. Mean values of predicted HC50 for all 10 chemicals are within the upper and lower bounds of experimental counterparts, regardless of the species and number of data points. Figure 3 shows the mean SSD curves for chemical *chlorpyrifos* (2921-88-2), as well as the upper and lower bounds according to 1,000 times of bootstrapping (in light colors) for both experimental (red) and predictive (blue) SSDs. The range of experimental and predictive SSD are mostly overlapped according to Fig. 3. The HC50 values of *chlorpyrifos* based on predictive SSD range from 0.23 to 4.57 μmol/L, and the experimental HC50 values range from 0.17 to 6.92 μmol/L. On both curves, fishes tend to be more sensitive to the exposure of *chlorpyrifos*. The species have the highest tolerance on the experimental SSD is *Sialis Lutaria* (Insects/Spiders) with LC50 61.66 umol/L, and on the predictive SSD is other water fleas with LC50 436.52 umol/L.

### Screening-level chemical ecotoxicity analysis

We applied our models to the organic chemicals in the ToX21 dataset. As a result, SSDs for 8424 organic chemicals were generated, among which 1240 fell into the AD for at least four ANN models (out of eight). Their predicted LC50 values, predictive HC5 and SSDs can be found in the supplementary information.

We mapped these chemicals with the ones registered as high-volume chemicals in European Chemicals Agency (ECHA) database (Registered substances-ECHA). We identified top 10 chemicals, of which the products of production volume and toxicity (inverse of HC5) are the highest (Table 3). If no additional data are available, these chemicals deserve attention given their high volume of usage and the high ecotoxicity, according to our screening-level analysis results (see IS for the full screening results). Among them 4,4′-diphenylmethane diisocyanate (101-68-8, MDI) shows the highest volume X toxicity value. MDI is widely used in the manufacture of polyurethane. MDI makes up about 60% of the global production of diisocyanate in 2000 (Randall and Lee 2002), and the U.S. demand for pure MDI was about 200 million pounds in 2008 (US EPA 2015b). Under certain circumstances, MDI can be released from adhesive and sealants in a format that isn’t completed reacted, therefore cause potential occupational exposure (US EPA 2015b).

**Table 3:** The top chemicals with the highest threatening among the registered chemicals in the ECHA database

Chemical name	Chemical CAS	HC5 umol/L	Number of chemicals in model AD	Production band in ECHA (thousand tonnes year^−1^)
4,4′-Diphenylmethane diisocyanate	101-68-8	0.19	4	100–1000
2-Ethylhexyl acrylate	103-11-7	3.1	4	100–1000
2-Ethylhexyl nitrate	27247-96-7	5.6	5	100–1000
Anthraquinone	84-65-1	0.13	4	1–10
tert-Butylperoxy 2-ethylhexyl carbonate	34443-12-4	0.19	4	1–10
Dodecanoic acid	143-07-7	2.6	4	10–100
2-Methyl-4′′′-(methylthio)-2-morpholinopropiophenone	71868-10-5	0.29	5	1–10
Methyl dodecanoate	111-82-0	3.3	4	10–100
6H-Dibenzo[c,e][1,2]oxaphosphinine 6-oxide	35948-25-5	0.37	5	1–10
1,3-Benzenedicarboxylic acid	121-91-5	57.1	4	100–1000

### OVL testing

SSDs can be fitted by different statistical distributions. We used the coefficient of overlapping (OVL) method to compare the performance of different statistical distributions: log-normal, Weibull and Gamma, when fitting SSD curves. As the results show, the average OVL score of log-normal distribution was 0.82. More than 93% of the 8424 SSDs have OVL score higher than 0.60 on log-normal distribution. The comparison between log-normal, Weibull and Gamma distributions is presented in Fig. S3. The average OVL scores for Weibull and Gamma distributions were 0.71 and 0.67, respectively. Log-normal distribution was the one that has the highest average OVL score among all distributions we tested. The resulting standard log-normal SSD function shows the average logmean (µ) and average GSD (geometric standard deviation, *σ*) of 3.21 and 2.58, respectively, for the 8424 SSDs.

## Discussion

To our knowledge, our study is the first that consolidated aquatic ecotoxicity data from multiple data sources, and used them for large-scale SSD development using ANN. The resulting dataset, which is, to our best knowledge, the largest of its kind, is made freely available through our website. The predictive SSD, can be used for screening analysis to estimate the safety concentration of chemicals in aquatic ecosystem. Our results can also be used for as the reference ecotoxicity data in LCIA when better quality data are lacking, which is a acknowledged problem in LCA (Reap et al. 2008).

The performance of QSAR models developed in this study was promising and the results were comparable tothe existing QSARs in literature (Buccafusco et al. 1981; Devillers 2001; Toropov 2017). Our study demonstrates that advanced machine learning models can be used to improve the performance of QSAR models.

We collected extensive chemical toxicity dataset from reputable databases including ECOTOX and eChem. The datasets collected include over 2000 data points with comparable experimental conditions for reliable QSAR modeling. The QSAR models developed in this study using ANN can achieve *R*^2^ > 0.7, when the training dataset is larger than 300 data points for a single species. With lesser data points, the ANN model must be reduced to simpler structure, which compromises its ability to estimate chemical toxicity. For future studies, even larger training dataset would be desirable to minimize the chance of missing out uncommon chemical structure–toxicity relationships.

We used the QSAR models to create predictive SSDs in this study. Our predictive SSDs showed a good performance compared with the SSDs created by experimental values. In Fig. 2, the predictive SSD showed lower toxicity at low concentration, and higher toxicity at high concentration. This is mainly due to the fact that the experimental SSD was created with less toxicity data points, compared with our predictive SSD. It is expected that the accuracy of SSD increases with more toxicity data points, and more diverse taxa (Posthuma et al. 2001, 2019). It is notable that the predictive SSD showed closer HC50 values compared with the experimental SSDs.

We recommend that the predictive model to be used as a supplementary to experimental data. Our models cannot replace SSDs derived from experimental toxicity data, as it has prediction uncertainties, and focusing on single stressor in this study. Furthermore, since ANN is a “black box” model, it should not be used to interpret the mechanism of ecotoxicity with molecular structure (Stojić et al. 2010). Given the current scarcity of experimental data and the high cost of developing them, however, we believe that our results demonstrate the potential for machine learning techniques to be used as a proxy for SSDs when better information is lacking. Furthermore, the rapidly growing number of chemicals in the lab and in the marketplace makes it challenging for experimental data alone to meet the needs for understanding the potential ecotoxicological impact of chemicals. We believe that our results can serve as a screening tool in the absence of experimental data to prioritize the candidates for further analysis. We view machine learning techniques not as a replacement of but as a complementary tool for experimental studies. Experimental toxicity studies are also crucial for improving the quality of machine learning models to follow. High species sensitivity or low HC5 values in our SSD database should constitute a reason for in-depth testing, although predicted low species sensitivity or high HC5 values alone should not be taken as a proof that the chemical is safe.

We believe that the complementarity between predictive modeling and experimental studies can be further improved by standardizing the conditions for toxicity experiments and reporting. First of all, we cannot emphasize enough the importance of standard data exchange protocol on experimental conditions which is critical to accommodate machine readability of experimental data. Due to the poor documentation and the lack of standard data exchange protocol, extracting data on experimental conditions from existing literature and databases required painstaking effort. Second, consistency in experimental methods is crucial. We could not utilize many valuable experimental data points because one or more experimental conditions were not identical to the rest of the dataset. The variation in experimental conditions in e.g., duration of exposure, temperature, and chemical purity, significantly degraded the value of experimental toxicity data. A wider adoption of standard protocols for documenting and sharing toxicity testing results is urgently needed to tap into and maximize the value of experimental toxicity data for predictive modeling. While there are existing standards and guidelines including the OECD Test Guidelines, the Good Laboratory Practice (GLP) principles, and the Catalogue of Standard Toxicity Tests for Ecological Risk Assessment (Epa 1994), a universal applicable testing guideline is still lacking.

Machine learning techniques for ecotoxicological applications are still in a nascent stage, and there is considerable room for improvement in our study. Experimental data of better quality and quantity will improve the performances of the ANNs. Our models do not properly represent the toxicological impacts under multi-stressor conditions, because the experimental data used for training our model are all based on single chemical species. In fact, mixtures of chemicals are scarcely tested for ecotoxicity, and the development of protocols for mixture testing and reporting is in its infancy. In reality, however, ecosystem species are exposed to multiple chemicals at any given time. Although there are some studies that evaluate the concentration addition effect of chemical mixture (Hermens et al. 1984; Broderius and Kahl 1985; Wolf et al. 1988; Niederlehner et al. 1998), given that the number of possible combinations of chemical mixtures in both composition and proportion is extremely large, experimental data alone won’t be able to meet the growing needs of data. Additional data and research are needed to adequately address the ecotoxicological impacts of multiple stressors, especially in the context of using SSDs.

## Supplementary Information

Below is the link to the electronic supplementary material.

Supplementary file1 (XLSX 212 kb)

Supplementary file2 (XLSX 1942 kb)

Supplementary file3 (PDF 1488 kb)

## Data Availability

All code in this study were developed in Python 2.7 (Anaconda2) on a machine with Ubuntu 16.04 LTS system. The code and the pretrained ANN models are available in GitHub: https://github.com/RunshengSong/QSAR_SSD_Toolbox. Instruction on how to reproduce the results in this study is also provided in the Github repository.
