# Supplementary material for: Accelerating the pace of ecotoxicological assessment using artificial intelligence
Source: Ambio. 2021 Aug 24;51(3):598–610. doi: 10.1007/s13280-021-01598-8 (PMC8800994; doi:10.1007/s13280-021-01598-8)
Supplement: Supplementary file 3 — Supplementary file3 (PDF 1488 kb) [file 13280_2021_1598_MOESM3_ESM.pdf]

Ambio

Supplementary Information

This supplementary information has not been peer reviewed.

Title: **Accelerating the pace of ecotoxicological assessment using artificial intelligence**

Authors:
Runsheng Song, DingshengLi, Alexander Chang, Mengya Tao, Yuwei Qin, Arturo A. Keller and Sangwon Suh

Experimental Data Collection Procedure

Experimental ecotoxicity data (LC50) of organic chemicals on 8 aquatic species was collected from major public databases, such as ECOTOX, eChem, EFSA and HSDB.[1–5] Data from peer-reviewed literatures was also added as supplementary data to develop the neural network models in this study.[6–20] The number of organic chemicals collected for 8 different species (in three taxa) is presented in Figure S1, along with the taxa information for these species.

To ensure data quality, the critical experimental conditions, such as testing duration, chemical purity and *pH* values were strictly controlled during the process of data collection. 96 hours LC50 data was used for all species except water fleas (48 hours' data was used). Chemical purity must be higher than 85%. And the *pH* value must be in the range of 5 to 9. Experimental data that not meet these requirements was discarded. For chemical with multiple experimental values, the geometric mean was used in the final dataset. The species selected in this study is aiming to cover as many aquatic taxa as possible but also should have enough experimental ecotoxicity data. After the data collection and selection, species with less than 100 unique organic chemicals' experimental values were discarded. However, to utilize some of the discarded data, experimental values that met our data selection procedure for other water fleas (*Ceriodaphnia Dubia, Daphnia Pulex and Mix Water Flea*) in ECOTOX database was combined and treated as an individual species in this study.

Additional information, such as the CAS number, SMILEs, molecular weight and the chemical names were also collected. The unit of the LC50 values were

converted to *log10(LC50)* in *μmol/L*. The final dataset is available in the supplementary information.

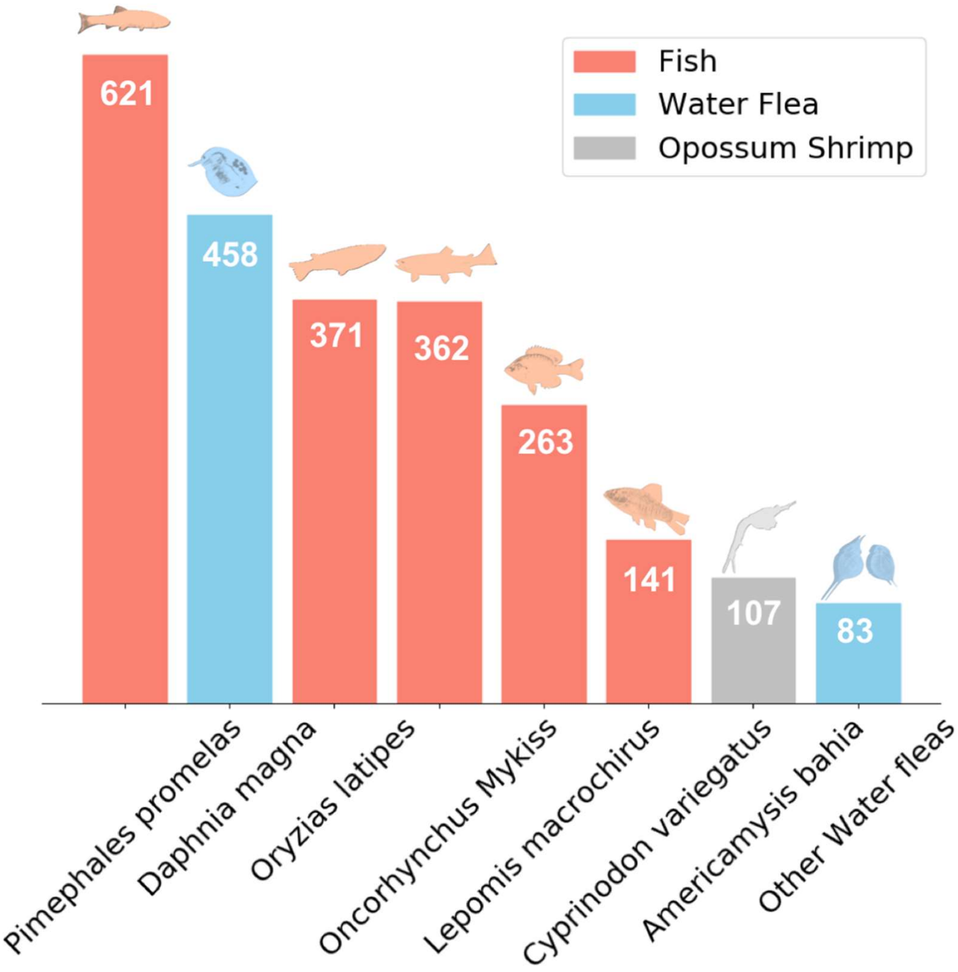

Figure S1. The number of unique chemicals collected for this study for 8 different species.

Model Performances and Hyperparameters

Table S1. The performances (in R^2) of the QSARs on testing dataset (20 randomly selected chemicals) along with the hyper-parameters optimized in this study. For all QSARs, Rectified Linear unit (ReLu) activation function was used in hidden neuron. Learning rate was set to 0.001. The number of training iteration was 500 times.

QSAR for Species	*Pimephales Promelas*	*Daphnia Magna*	*Oryzias Latipes*	*Oncorhynchus Mykiss*	*Lepomis Macrochirus*	*Cyprinodon Variegatus*	*Americamysis Bahia*	Other Water Fleas
Model Performance (R^2) on Testing Data	0.71	0.75	0.54	0.75	0.72	0.66	0.67	0.63
Number of Hidden Layer	2	1	2	2	2	2	1	2
Number of Hidden Neuron in Each Layer	32 × 16	16	64 × 32	64 × 32	32 × 16	16 × 8	16	16 × 8
Activation Functions	ReLu, ReLu	ReLu	ReLu, ReLu	ReLu, ReLu	ReLu, ReLu	ReLu, ReLu	ReLu	ReLu, ReLu
Regularization Factor	0.01	0.02	0.01	0.02	0.03	0.05	0.01	0.05

Screening the ToX21 Database

Table S2. The top 10 chemicals among the chemicals in the ToX21 database with the lowest HC5 values according to the predictive SSDs.

Chemical Name	CAS Number	HC5 values (log(umol/L)
Dihydrostreptomycin sulfate	5490-27-7	-38.6184
Streptomycin sulfate (2:3)	3810-74-0	-37.9823
Netilmicin sulfate	56391-57-2	-36.2539
Sisomicin sulfate	53179-09-2	-33.8234
Sucrose octasulfate-aluminum complex	54182-58-0	-25.4343
Triptorelin pamoate	124508-66-3	-23.3853
YM218		-21.9758
Ergotamine D-tartrate	379-79-3	-20.7683
Pyrvinium pamoate	3546-41-6	-19.7536
Auranofin	34031-32-8	-18.8429

Model Applicable Domains

Table S3. The results of model AD analysis for each QSAR in this study. The cut-off threshold determines whether chemicals fall inside or outside a model's AD base on its distance to the centriole of the training data. The average mean square errors (MSEs) of the chemicals in the testing data that are inside and outside the AD of each model are also reported in the table.

QSAR for Species	AD Cut-off Threshold (K)	Average MSE Inside AD	Average MSE Outside AD
Pimephales Promelas	3	8%	220%
Daphnia Magna	2.5	7%	12%
Oryzias Latipes	1.5	8%	19%
Oncorhynchus Mykiss	1	6%	15%
Lepomis Macrochirus	1	6%	22%
Cyprinodon Variegatus	2.5	7%	16%
Americamysis Bahia	2	17%	19%
**Other Water Fleas**	3	22%	32%

Comparing Predictive SSDs with Experimental SSDs

Table S4a. The predictions of the ANN models for the 10 selected chemicals. The unit is log(μmol/L)

	**Clofenotane**	**Pentachlorophenol**	**Lindane**	**Propiconazole**	**Imidacloprid**	**Endosulfan**	**Chlorpyrifos**	**Fluoranthene**	**Aniline**	**Diazinon**
	**50-29-3**	**87-86-5**	**58-89-9**	**60207-90-1**	**138261-41-3**	**115-29-7**	**2921-88-2**	**206-44-0**	**62-53-3**	**333-41-5**
Americamysis Bahia	-2.8529	-0.2589	-0.6759	-0.2245	0.8985	-2.0640	-1.3199	-0.9427	2.0983	-2.2168
Lepomis Macrochirus	-1.2054	-0.0014	0.2492	-0.0044	1.6714	-1.6125	-0.6156	0.4946	2.1022	-0.9079
Oncorhynchus Mykiss	-1.1660	0.0241	0.4198	0.1999	1.7069	-1.1038	-0.5483	0.7428	2.3500	-0.7943
Cyprinodon Variegatus	-0.7202	0.2318	0.8196	0.2381	1.7209	-0.3951	-0.3919	0.7745	2.4209	0.5804
Daphnia Magna	-0.6343	0.3607	0.9857	0.4997	1.8418	0.1452	-0.1502	0.9271	2.4288	0.6645
Pimephales Promelas	0.0526	0.4146	1.7567	0.5351	1.8915	0.2450	-0.1034	1.0595	2.4303	0.8129
Oryzias Latipes	0.3035	0.6400	2.4230	1.3133	2.5427	0.5154	0.8825	1.0844	2.5558	0.8664
Other water fleas	2.6481	1.1353	4.2927	2.5075	4.4979	0.6675	2.0752	2.9870	3.4299	1.7595

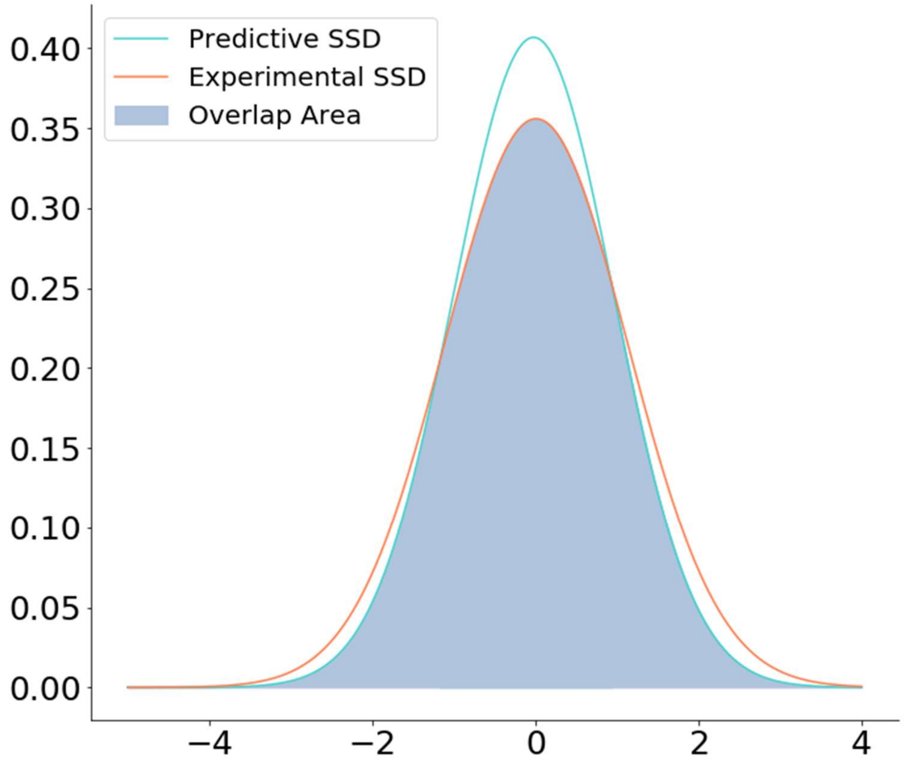

Figure S2. The predictive SSD (in PDF format), the experimental SSD and the overlapping area for chemical chlorpyrifos (2921-88-2). The OVL score is 92%.

OVL Testing for SSD Fitting

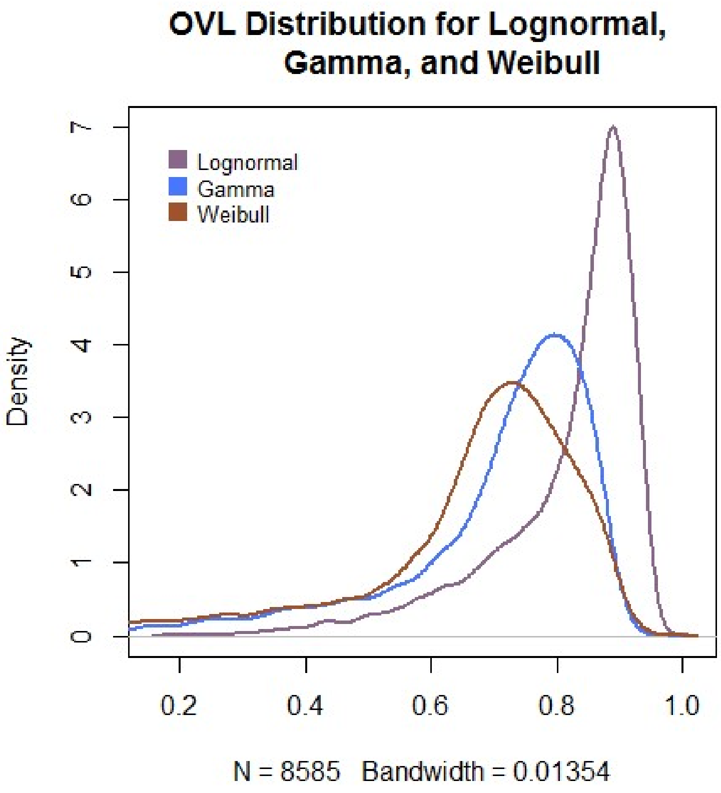

Figure S3. Comparison between log-normal, Weibull and Gamma distributions in OVL testing for the SSDs of the ToX21 chemicals.

Table S5. OVL scores for Log-normal, Gamma and Weibull distributions.

Average OVL		
Lognormal	Gamma	Weibull
81.7%	70.8%	67.2%

The Descriptors Used to Develop ANN Models for Each Species

Table S6. The full list of descriptors used to develop each ANN model. The full name of each descriptors are listed in Table S7.

Pimephales Promelas	Daphnia Magna	Oryzias Latipes	Oncorhynchus Mykiss	Lepomis Macrochirus	Cyprinodon Variegatus	Americamysis Bahia	Other Water Fleas

SLogP	SLogP	SLogP	SLogP	SLogP	SLogP	SLogP	SLogP
Xp-2dv	Xp-2dv	Xp-2dv	AATS3i	ATS1m	SMR_VSA4	PEOE_VSA6	MWC03
PEOE_VSA6	SM1_Dzm	PEOE_VSA6	ATS0m	ATS2m	SM1_Dzm	SMR_VSA4	AATS5v
Sm	Xp-4dv	Sm	ATSC2p	ATS3m	Xp-4dv	AATS8i	AATS6i
AATS0i	MWC03	AATS3v	ETA_eta	ATS4m	ATS1m	ATSC1m	ATSC1dv
ATS0p	AATS6v	BertzCT	MWC05	C3SP3	ATSC2m	ATSC3v	ATSC1m
ATS3m	ATS2m	nCl	SlogP_VSA4	NssO	ATSC4dv	ATSC6m	ATSC5dv
ATS5m	ATS3m	PEOE_VSA1	Xc-3d	Xp-3dv	ETA_eta	C3SP2	ETA_alpha
piPC3	ATS5m	WPath		Xp-5dv	GGI1	nRot	MAXdO
VR3_Dzi	BCUTv-1h	Xp-0dv			MID_N	SlogP_VSA11	piPC7
ZMIC2	EState_VSA8	ZMIC2			PEOE_VSA3	SMR_VSA9	Xpc-4dv
	IC4				SpAbs_D	Xpc-4dv	ZMIC4
	MPC5				SRW05	ZMIC2	
	SlogP_VSA11				Xpc-4d		
	Zagreb1				Xpc-4dv		

Table S7. The full name of the descriptors used in Table S6.

Abbreviation	Full Name
**SLogP**	Wildman-Crippen LogP
**Xp-2dv**	2-ordered Chi path weighted by valence electrons
**PEOE_VSA6**	MOE Charge VSA Descriptor 6 (-0.10 <= x < -0.05)
**Sm**	sum of constitutional weighted by mass
**AATS0i**	averaged moreau-broto autocorrelation of lag 0 weighted by ionization potential
**ATS0p**	moreau-broto autocorrelation of lag 0 weighted by polarizability
**ATS3m**	moreau-broto autocorrelation of lag 3 weighted by mass
**ATS5m**	moreau-broto autocorrelation of lag 5 weighted by mass
**piPC3**	3-ordered pi-path count (log scale)

**VR3_Dzi**	logarithmic Randic-like eigenvector-based index from Barysz matrix weighted by ionization potential
**ZMIC2**	2-ordered Z-modified information content
**SM1_Dzm**	spectral moment from Barysz matrix weighted by mass
**Xp-4dv**	4-ordered Chi path weighted by valence electrons
**MWC03**	walk count (leg-3)
**AATS6v**	averaged moreau-broto autocorrelation of lag 6 weighted by vdw volume
**ATS2m**	moreau-broto autocorrelation of lag 2 weighted by mass
**BCUTv-1h**	first heighest eigenvalue of Burden matrix weighted by vdw volume
**EState_VSA8**	EState VSA Descriptor 8 (2.05 <= x < 4.69)
**IC4**	4-ordered neighborhood information content
**MPC5**	5-ordered path count
**SlogP_VSA11**	MOE logP VSA Descriptor 11 (0.50 <= x < 0.60)
**Zagreb1**	Zagreb index (version 1)
**AATS3v**	averaged moreau-broto autocorrelation of lag 3 weighted by vdw volume
**BertzCT**	Bertz CT
**nCl**	number of Cl atoms
**PEOE_VSA1**	MOE Charge VSA Descriptor 1 (-inf < x < -0.30)
**WPath**	Wiener index
**Xp-0dv**	0-ordered Chi path weighted by valence electrons
**AATS3i**	averaged moreau-broto autocorrelation of lag 3 weighted by ionization potential
**ATS0m**	moreau-broto autocorrelation of lag 0 weighted by mass
**ATSC2p**	centered moreau-broto autocorrelation of lag 2 weighted by polarizability
**ETA_eta**	ETA composite index for reference graph
**MWC05**	walk count (leg-5)
**SlogP_VSA4**	MOE logP VSA Descriptor 4 (0.00 <= x < 0.10)
**Xc-3d**	3-ordered Chi cluster weighted by sigma electrons
**ATS1m**	moreau-broto autocorrelation of lag 1 weighted by mass
**ATS4m**	moreau-broto autocorrelation of lag 4 weighted by mass
**C3SP3**	SP3 carbon bound to 3 other carbons
**NssO**	number of ssO
**Xp-3dv**	3-ordered Chi path weighted by valence electrons
**Xp-5dv**	5-ordered Chi path weighted by valence electrons
**SMR_VSA4**	MOE MR VSA Descriptor 4 (2.24 <= x < 2.45)
**ATSC2m**	centered moreau-broto autocorrelation of lag 2 weighted by mass
**ATSC4dv**	centered moreau-broto autocorrelation of lag 4 weighted by valence electrons
**GGI1**	1-ordered raw topological charge
**MID_N**	molecular ID on N atoms
**PEOE_VSA3**	MOE Charge VSA Descriptor 3 (-0.25 <= x < -0.20)

**SpAbs_D**	graph energy from distance matrix
**SRW05**	walk count (leg-5, only self returning walk)
**Xpc-4d**	4-ordered Chi path-cluster weighted by sigma electrons
**Xpc-4dv**	4-ordered Chi path-cluster weighted by valence electrons
**AATS8i**	averaged moreau-broto autocorrelation of lag 8 weighted by ionization potential
**ATSC1m**	centered moreau-broto autocorrelation of lag 1 weighted by mass
**ATSC3v**	centered moreau-broto autocorrelation of lag 3 weighted by vdw volume
**ATSC6m**	centered moreau-broto autocorrelation of lag 6 weighted by mass
**C3SP2**	SP2 carbon bound to 3 other carbons
**nRot**	rotatable bonds count
**SMR_VSA9**	MOE MR VSA Descriptor 9 (3.80 <= x < 4.00)
**AATS5v**	averaged moreau-broto autocorrelation of lag 5 weighted by vdw volume
**AATS6i**	averaged moreau-broto autocorrelation of lag 6 weighted by ionization potential
**ATSC1dv**	centered moreau-broto autocorrelation of lag 1 weighted by valence electrons
**ATSC5dv**	centered moreau-broto autocorrelation of lag 5 weighted by valence electrons
**ETA_alpha**	ETA core count
**MAXdO**	max of dO
**piPC7**	7-ordered pi-path count (log scale)
**ZMIC4**	4-ordered Z-modified information content
